# Autoimmune Encephalitis With Psychotic Manifestations and Cognitive Impairment Presenting as Schizophrenia: Case Report and Literature Review

**DOI:** 10.3389/fpsyt.2022.827138

**Published:** 2022-02-14

**Authors:** Yuanyuan Luo, Jieying Li, Fugui Jiang, Arui Tan, Xiaohong Qin, Xiaoqiang Xiao, Zuxing Wang, Peijia Wang, Yang Yi, Juan Li, Shuai Yuan, Lei Liu, Jun Xiao

**Affiliations:** ^1^Sichuan Provincial Center for Mental Health, Sichuan Academy of Medical Science & Sichuan Provincial People's Hospital, Chengdu, China; ^2^Key Laboratory of Psychosomatic Medicine, Chinese Academy of Medical Sciences, Chengdu, China; ^3^The Fourth People's Hospital of Chengdu, Chengdu, China; ^4^Department of Neurology, Beijing Tongren Hospital, Capital Medical University, Beijing, China

**Keywords:** autoimmune encephalitis, anti-AMPAR encephalitis, anti-NMDAR encephalitis, schizophrenia, psychotic

## Abstract

Autoimmune encephalitis is characterized by mental and behavioral symptoms, seizures, and cognitive impairment. The presence of schizophrenia needs to be distinguished from that of autoimmune encephalitis. Herein, we describe the case of a woman who exhibited abnormal mental behavior and cognitive impairment. The patient had experienced similar symptoms more than 20 years previously and had been diagnosed with schizophrenia. The patient's psychotic symptoms improved after treatment with antipsychotic drugs; however, cognitive impairment persisted. She was diagnosed with anti-N-methyl-D-aspartate (NMDA)-receptor concurrent with anti-α-amino-3-hydroxy-5-methyl-4-isoxazolepropionic acid (AMPA)-receptor encephalitis. She showed improvement after treatment with steroids and intravenous immunoglobulins (IVIgs). Furthermore, we reviewed the literature and found that, including the present case, 10 patients have been diagnosed with anti-NMDA concurrent with anti-AMPA-receptor encephalitis. Three of these patients were men and seven were women, and their ages ranged from 21 to 71 years. Moreover, seven (70%) patients had a history of tumors. Symptoms of these patients included psychotic symptoms, varying degrees of consciousness disturbance, seizures, dyskinesia, dystonia, autonomic dysfunction, agitation, and verbal reduction. Brain magnetic resonance imaging findings showed scattered fluid-attenuated inversion recovery hyperintensity in subcortical white matter and/or medial temporal lobe in seven (70%) patients. After combination treatment, including tumor removal and administration of steroids, IVIg, plasma exchange, or immunity inhibitors, the symptoms improved in part of the patients. It is necessary to exclude autoimmune encephalitis for patients with psychiatric manifestations and cognitive impairment. Timely combination therapy is important in anti-NMDA-receptor concurrent with anti-AMPA-receptor encephalitis.

## Introduction

Autoimmune encephalitis is associated with antibodies against neuronal cell-surface or synaptic proteins, including neurotransmitter receptors; this disorder can develop with core symptoms that are similar to those of infectious encephalitis, including mental symptoms, cognitive impairment, and epilepsy (1, 2). Some neuronal surface antigens associated with this condition have been identified as follows: N-methyl-D-aspartate (NMDA) receptor, α-amino-3-hydroxy-5-methyl-4-isoxazolepropionic acid (AMPA) receptor, metabotropic glutamate receptor, contactin-associated protein-like 2 (Caspr2), gamma-aminobutyric acid-B (GABAb) receptor, glycine receptor, leucine-rich glioma-inactivated 1 (LGI1) (3, 4). The symptoms caused by these antibodies are similar to those of schizophrenia, dementia, and sleep disorders (5–7).

NMDA, AMPA, and metabotropic glutamate receptors are the major excitatory neurotransmitter glutamate receptors of the central nervous system. NMDA receptors are ligands and voltage-gated ionotropic receptors that promote the influx of Ca^2+^ and Na^+^ (8, 9) and play a key physiological role in synaptic functions such as synaptic plasticity, learning, and memory (10). Patients with anti-NMDA-receptor encephalitis present with abnormal (psychiatric) behaviors or cognitive dysfunction; speech dysfunction (pressured speech, verbal reduction, or mutism); seizures; movement disorders, dyskinesia, rigidity, or abnormal postures; decreased level of consciousness; and autonomic dysfunction or central hypoventilation (10, 11). In addition, AMPA-type ionotropic glutamate receptors, which are the major brain excitatory neurotransmitter receptors, are tetrameric ligand-gated ion channels comprising GluA1-4 subunits (12). Patients with anti-AMPA-receptor encephalitis mainly present with short-term memory loss, confusion, mood disturbances, sleep disorders, seizures, and psychosis (13, 14).

Previous studies have found that patients with no or minimal neurological features who have tested positive for neuronal autoantibodies show sufficient differences from typical autoimmune encephalitis and have proposed a novel category of autoimmune psychosis (15, 16). Pollak et al. proposed a novel and conservative approach to recognize autoimmune psychosis (16). However, it was found that the criteria for autoimmune psychosis have limited utility in the absence of neurological symptoms or when paraclinical test results are normal (17). Another study had revealed that anti-NMDA-receptor encephalitis as defined by consensus criteria rarely occurred in psychiatric patients (18). In addition, there were no differences between seropositive and seronegative anti-NMDA-receptor antibodies patients in the score improvement for Positive and Negative Syndrome Scale after completing 4 weeks of amisulpride treatment (19). Therefore, further studies are required to identify the characteristics of autoimmune psychosis.

Psychotic manifestations are often observed among patients with autoimmune disorders (20, 21). Anti-NMDA-receptor encephalitis might be present in a subset of patients with psychosis, and immunotherapy can be considered a treatment option for patients who fail to respond to other therapies (22). Moreover, although pure psychotic manifestations of antibody-associated autoimmune encephalitis without any additional neuropsychiatric findings are rare (23), it is easy to overlook autoimmune encephalitis, particularly when the neurological symptoms are atypical. In this study, we present our experience with a patient with autoimmune encephalitis who was initially diagnosed with schizophrenia and also review previous cases.

## Case Presentation

**Figure 1:**
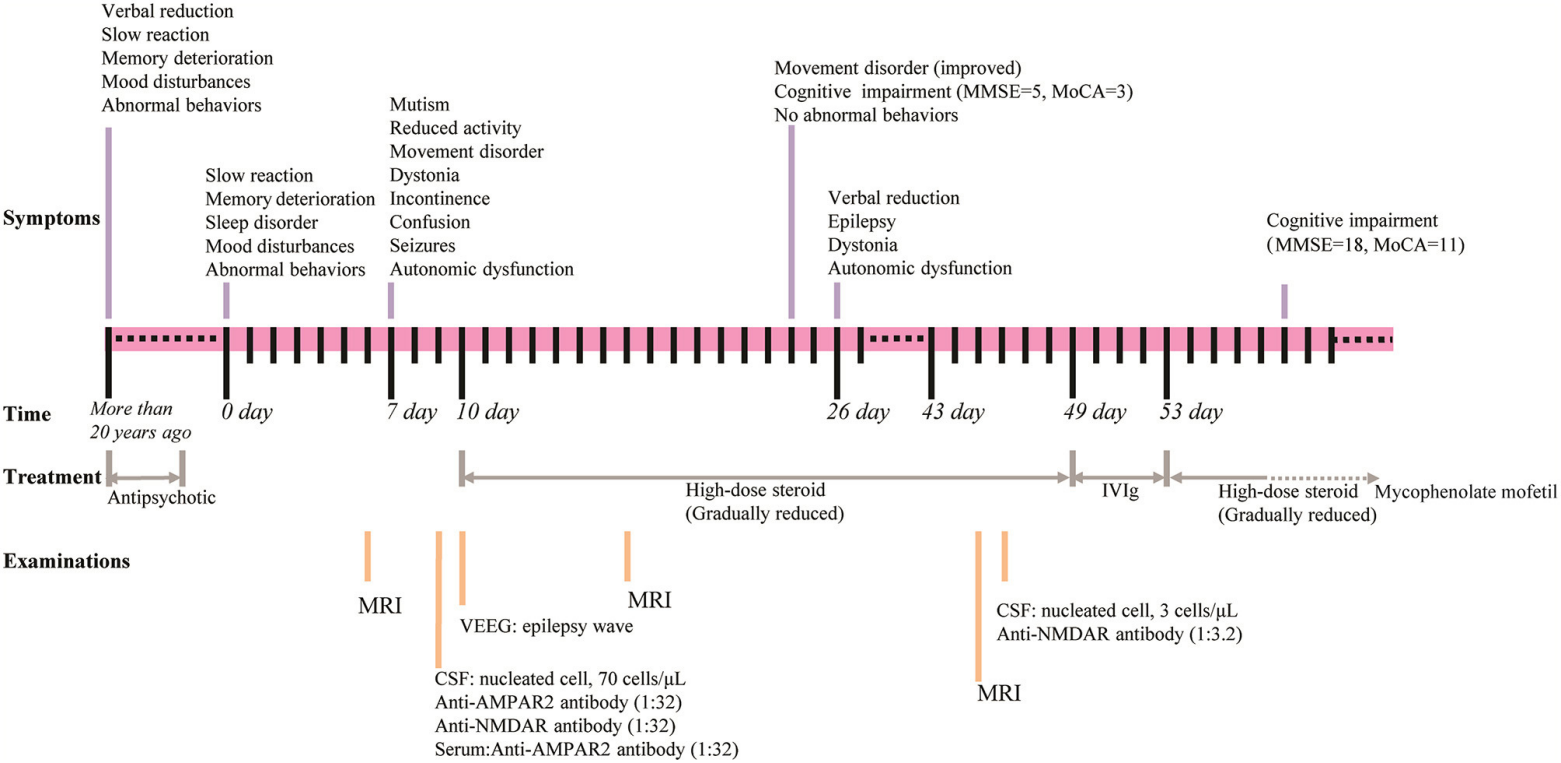
Timeline for the course of the case. Upper part shows the symptoms, middle shows the time and the treatment, and lower part shows the examinations conducted.

A 50-year-old woman presented to the hospital with slow reaction, memory deterioration, sleep disorder, mood disturbances, and abnormal behaviors including repetitive stereotyped words and behaviors, decreased social interaction, and incontinence (Figure 1). The patient's condition worsened before the diagnosis of encephalitis was made. Worsening was indicated by mutism, reduced activity, movement disorder, dystonia, incontinence, confusion, seizures, and autonomic dysfunction. The modified Rankin Scale (mRS) (24, 25) score was 4. Notably, the patient had experienced similar symptoms >20 years previously and was diagnosed with schizophrenia. The diagnostic criteria referred to International Classification of diseases. The main symptoms previously were mood disturbances (anxiety and fear), abnormal behaviors (repetitive stereotyped behaviors, decreased social interaction, and catatonic stupor), verbal reduction, slow reaction, and memory deterioration, and after treatment with antipsychotic drugs, the patient's mental symptoms improved, but persistent cognitive impairment was noted. In addition, the patient had a history of a hydatidiform mole that was surgically removed 15 years previously.

**Figure 2:**
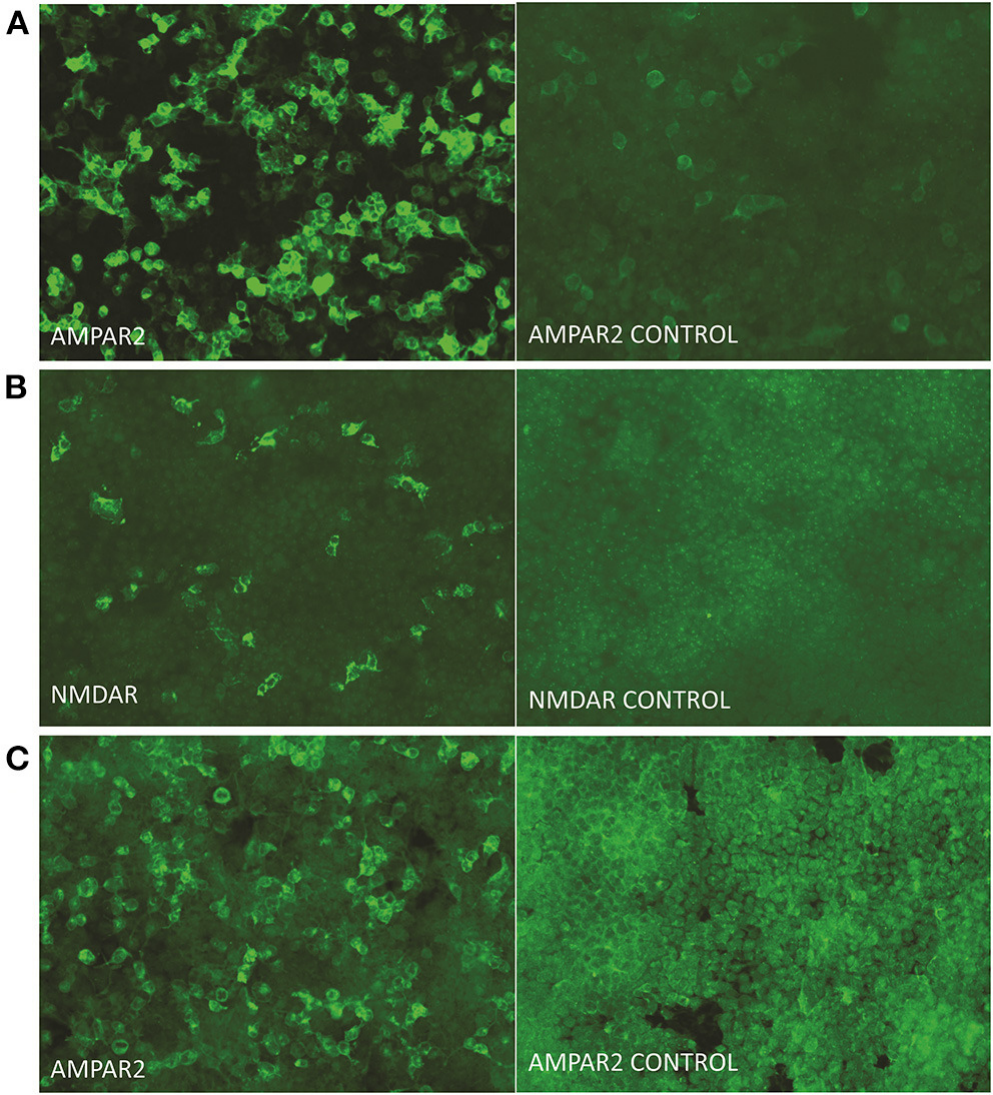
Presence of autoimmune antibodies in serum and CSF. Immunoactivity of the anti-AMPA-receptor 2 antibody **(A)**, anti-NMDA-receptor antibody **(B)** in cerebrospinal fluid, and anti-AMPA-receptor 2 antibody in serum **(C)** at 9 days after onset. The titer of antibodies in the cerebrospinal fluid and serum were measured as 1:32 (original magnification, 200 ×).

Blood test results showed normal levels of antinuclear antibodies and comprehensive metabolism. The presence of tumors was ruled out via chest and abdomen computed tomography and breast ultrasound. Video electroencephalography showed an epilepsy wave. Initial cerebrospinal fluid (CSF) study revealed that lymphocyte count increased with normal protein (total protein, 0.478 g/L; glucose, 3.77 mmol/L; chlorine 127.0 mmol/L; nucleated cell at 70 cells/μL including 8% neutrophils and 85.2% lymphocytes). Infection studies revealed negative results for cryptococcus and tuberculosis as well as other bacteria and fungi. Investigation for autoimmune encephalitis was performed by collecting antibodies, including LGI1 protein, GABAb receptor, NMDA receptor, Caspr2, AMPA1 receptor, AMPA2 receptor, Hu, Yo, Ri, CV2, Ma2, and amphiphysin from the serum and CSF. Anti-AMPA receptor 2 (1:32) (Figure 2A) and anti-NMDA receptor (1:32) (Figure 2B) antibodies were detected in the CSF, and anti-AMPA receptor 2 (1:32) was detected in serum (Figure 2C).

**Figure 3:**
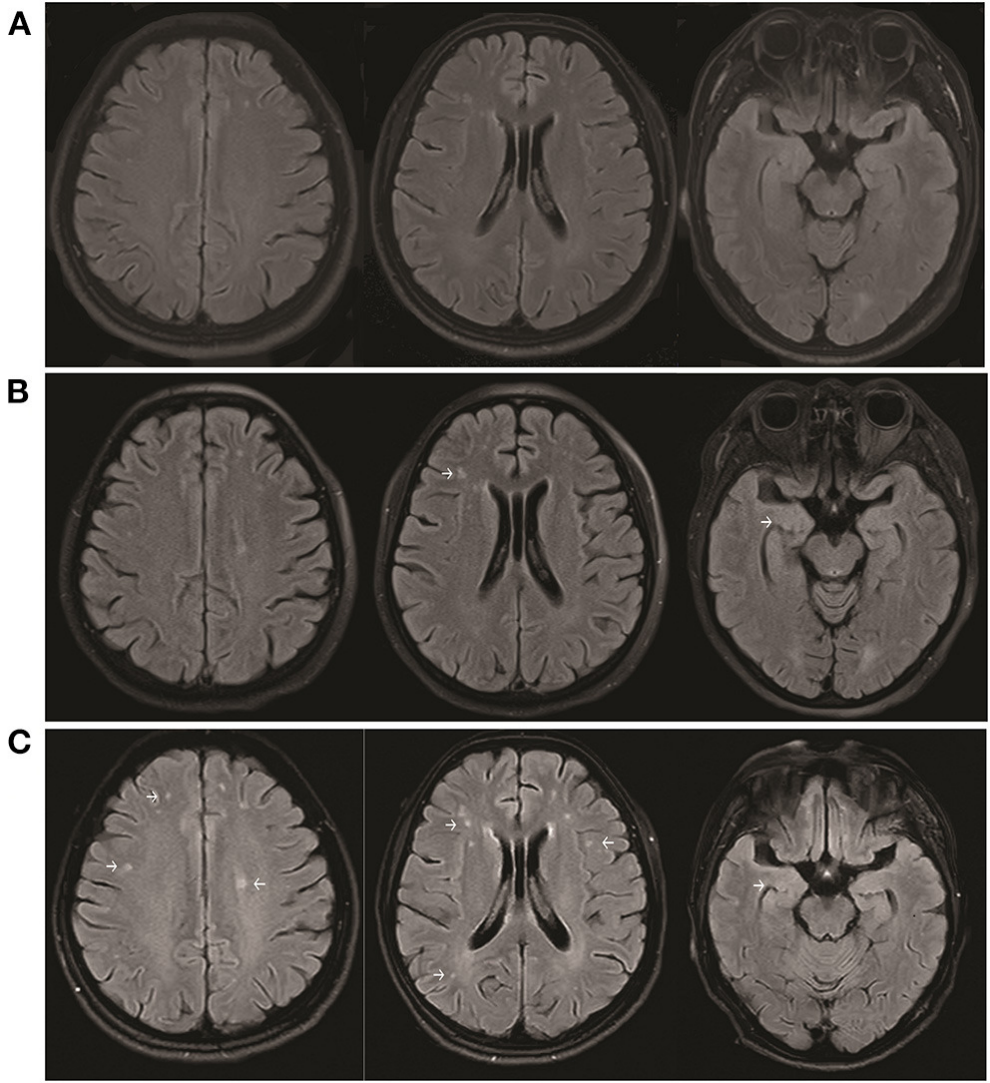
Brain MRI showing increased scattered fluid-attenuated inversion recovery hyperintensity in the subcortical white matter and medial temporal lobe at 17 days **(B)** and 45 days **(C)** after onset compared with 6 days **(A)** after onset.

The patient was started on high-dose steroid therapy on the 10th day after onset, and the dose was subsequently and gradually reduced (Figure 1). After 1 month, her cognitive function (total scores on Mini-mental State Examination and Montreal Cognitive Assessment were 5 and 3, respectively) and movement disorder were partially improved. Abnormal behaviors were not observed, although the patient still showed verbal reduction, epilepsy, dystonia, and autonomic dysfunction. Brain magnetic resonance imaging (MRI) revealed that scattered fluid-attenuated inversion recovery (FLAIR) hyperintensity had increased in the subcortical white matter and medial temporal lobe at 17 days (Figure 3B) and 45 days (Figure 3C) after onset compared with 6 days (Figure 3A) after onset. Moreover, repeated CSF analysis revealed that anti-NMDA receptor antibody (1:3.2) was positive but anti-AMPA1 and anti-AMPA2 receptor antibodies were negative. Thereafter, a combination treatment including intravenous immunoglobulin (IVIg) at 2 g/kg for 5 days, steroids, and a second-line drug (mycophenolate mofetil) was administered. At the last follow-up, cognitive function was improved (total scores on Mini-mental State Examination and Montreal Cognitive Assessment were 18 and 11, respectively), and no abnormal mental symptoms, dystonia, seizures, and autonomic dysfunction were noted. The patient's mRS score was 2. The disease did not recur during the 30 weeks of follow-up.

## Review of Previously Reported Cases of Anti-AMPA Concurrent With Anti-NMDA-Receptor Encephalitis

To evaluate the clinical characteristics and outcome of anti-AMPA concurrent with anti-NMDA-receptor encephalitis in the clinical setting, we reviewed the previously reported studies (13, 26, 27). Including our patient, 10 patients with anti-AMPA-receptor concurrent with anti-NMDA-receptor encephalitis have been reported, but only 4 had complete clinical data (Table 1). Three patients were men and seven were women, giving a men-to-women ratio of 3:7. The ages of the patients ranged from 21 to 71 years. Seven (70%) patients showed abnormal brain MRI, eight (80%) had tumors, and one had a hydatidiform mole. All patients had varying degrees of consciousness disturbance (100%). Four patients for whom complete clinical data were available had seizures (100%), three had dyskinesia (75%), three had dystonia (75%), two had psychiatric symptoms (50%), two showed verbal reduction (50%), one had autonomic dysfunction and agitation, and one required intubation. After combined treatment, which included tumor resection and administration of steroids, IVIg, plasma exchange, cyclophosphamide, infusion of rituximab, or another second-line options, three patients showed complete response to the treatment, six patients showed partially response, and one patient died of neuroblastoma.

**Table 1:** Clinical features of patients with anti-AMPA-receptor concurrent with anti-NMDA-receptor encephalitis.

**No**.	**Age/sex**	**Sympton onset until diagnosis (week)**	**Clinical presentation**	**MRI**	**EEG**	**CSF**	
1	25/F	2	Psychosis, confusion, agitation, verbal reduction, seizures, dyskinesias, fever, hypertension, required intubation	Normal	NA	Normal WBC and protein	
2	71/M	5	Somnolent, seizures, disoriented, tremor	Abnormality in the hypothalamic region with mass effect on pituitary gland; T2/FLAIR increased signal in the right temporal lobe	Generalized slowing	Normal WBC, elevated protein	
3	50/F	1	Mutism, reduced activity, movement disorder, dystonia, incontinence, confusion, seizures, autonomic dysfunction	FLAIR hyperintensity scattered within subcortical white matter, medial temporal lobe	Epilepsy wave	Nucleated cell, 70 cells/μL, including 8% neutrophils, 85. 2% lymphocytes	
4	30/M	NA	Difficulty walking, seizures, unarousable, increased spasticity, neuropsychiatric abnormalities	Brain MRI revealed FLAIR hyperintensity scattered within mostly the subcortical white matter as well as an enhancing lesion adjacent to the left caudate nucleus	NA	WBC 97 (95% lymphocytes), protein 70 mg/dL, and glucose 65 mg/dL	
5-10^*^	(21-61)/5F, 1M	NA	Severe symptoms of anti-NMDA receptor encephalitis, consciousness level of all patients decreased, and required intensive care. 1 case had ocular muscle weakness.	4 Pts: bilateral medial temporal lobes	NA	NA	
**No**.	**Antibodies**	**Tumor**	**Symptom onset until start of treatment (week)**	**Neurologic outcome measuring mRS (compared with mRS at diagnosis)**	**Therapy**	**Treatment response**	**Follow-up, (week)**
1	AMPAR, NMDAR	Ovarian teratoma	2	0 (5)	Tumor resection, steroids, IVIg	Full	50
2	AMPAR, NMDAR	Thymic carcinoid	3	1 (4)	Tumor resection, steroids, plasma exchange	Full	78
3	AMPAR, NMDAR	Hydatid mole	1.5	2 (4)	IVIg, steroide, second line	Full	30
4	AMPAR, VGKCR, NMDAR	Thymoma	NA	NA	Tumor resection, steroids, IVIg, cyclophosphamide, infusion of rituximab	Part	8
5-10^*^	AMPAR, NMDAR	4 Pts: ovarian teratoma; 1 breast cancer; 1 GABAbR-Ab	NA	5 Pts: mRS score 4(0–6); 1 died of neuroblastoma	6 Pts: first line; 4 Pts: second line; 5 Pts tumor removal	NA	NA

* *The 5–10 cases did not have complete data*.

*F, female; M, male; WBC, white blood cell; Pts, patients; FLAIR, fluid-attenuated inversion recovery; mRS, modified Rankin Scale; IVIg, intravenous immunoglobulins*.

## Discussion

Autoimmune encephalitis is associated with many disorders and has been detected in patients with Alzheimer's disease (6), schizophrenia (5), bipolar disorder (28), and depression (29). Previous studies found that multiple antibodies, including glial and neuronal surface antibodies, can appear at the same time in patients with autoimmune encephalitis (26). Our patient presented with cognitive impairment, mood disturbances, speech dysfunction, sleep disorder, dystonia, autonomic dysfunction, and epilepsy. Moreover, the patient's CSF test was positive for anti-NMDA-receptor concurrent with anti-AMPA-receptor antibodies. The patient's symptoms improved after combination treatment with steroids and IVIg. Surprisingly, the patient had shown similar symptoms previously, resulting in her being diagnosed with schizophrenia. Her symptoms alleviated after treatment with antipsychotic drugs, but cognitive impairment and mood disorder were still noted.

Previous studies indicated that patients diagnosed with bipolar disorder, autistic traits, or psychotic disorder presented with autoimmune encephalitis a few years after the original diagnosis (20, 22, 28, 30), demonstrating that autoimmune encephalitis with prevalent psychiatric manifestations may be misdiagnosed and mistreated. Anti-NMDA-receptor encephalitis frequently has a relapsing course (30–32). During recovery, patients still present with alterations in behavior, memory, cognition, and executive functions (33). However, anti-AMPA-receptor encephalitis results in rapid deterioration of neurological function (14, 34). Research has revealed that anti-NMDAR IgA antibody in the brain likely interferes with neuronal function without causing acute neuronal degeneration or inflammatory changes and results in slow progressive cognitive impairment (35). Therefore, it is speculated that the patient may had anti-NMDA-receptor encephalitis but not anti-AMPA-receptor encephalitis >20 years previously. Patients with acute onset, atypical course of illness, neuropsychiatric symptoms, cognitive impairment, and rapid deterioration should be screened for autoimmune encephalitis (28, 36).

Ionotropic glutamate receptors are integral membrane proteins with a tetrameric structure containing four discrete semiautonomous domains: the extracellular amino-terminal, extracellular ligand-binding, transmembrane, and intracellular carboxyl-terminal domains (12). Sequences are similar among all glutamate receptor subunits, including AMPA, kainate, NMDA, and δ receptor (12). The antigens on the surface of the same cell can be simultaneously or successively affected when the immune system is abnormal (37). Jones et al. displayed a model of active immunization exists which developed a fulminant encephalitis; which is characterized by B-cell infiltrates, plasma cells, microglial activation, CD4^+^ T cells, rare neuronal loss, and antibodies against several epitopes of the GluN1 and GluN2 subunits of the NMDA receptor (38). Therefore, we speculated that receptors with the same structure might be affected at the same time in abnormal immune environments. Tumors and herpes simplex encephalitis can result in abnormal immunity (39, 40). The pathogenesis of autoimmune encephalitis is related to herpes simplex virus infection and tumors such as lung cancer, breast cancer, ovarian teratoma, and thymic cancer (3, 26, 41). Tumors often show anti-AMPA receptors and other additional antibodies at the same time (13). Literature review also showed that 8 of 10 patients had tumors. Therefore, it is necessary to screen tumors when two antibodies are identified.

AMPA receptor mediates most of the fast excitatory synaptic transmission in the brain and is important for synaptic plasticity, memory, and learning (42, 43). Anti-AMPA-receptor encephalitis manifests as limbic encephalitis with short-term memory loss, confusion, mood disturbances, epilepsy, psychosis, and sleep disorders (7, 13). Patients with anti-AMPA-receptor encephalitis may exhibit rapid deterioration of neurological function and present with additional antibodies (14, 34). Höftberger et al. revealed that the coexistence of onconeuronal antibodies predicted a poor outcome (13). AMPA receptors-dependent depolarization can remove the inhibition of NMDA receptors which are blocked by Mg^2+^ at resting potential (12), and NMDA receptors act as coincident detectors that sense postsynaptic depolarization at the same time or shortly after the release of glutamate or other excitatory amino acids. This process allows NMDA receptors to mediate cellular mechanisms of learning and memory (8, 12). We speculated that this may be the reason for the rapid deterioration of our patient's condition.

AMPA and NMDA receptors distributed in multiple areas of the brain, and the receptors in the corresponding parts showed corresponding symptoms. The presence of NMDA receptors in the presynapse is observed in several areas of the brain, such as the cortex, hippocampus, cerebellum, and spinal cord (9). AMPA receptors are broadly distributed in the cortex, hippocampus, cerebellum, basal ganglia (44) and peripheral nervous system (45). Previous studies displayed that the brain MRI in anti-AMPA and anti-NMDA-receptor encephalitis revealed a scattered FLAIR hyperintensity within mostly the subcortical white matter (27) and temporal lobe (13), which were consistent with our patient.

Early treatment and no admission to an intensive care unit were identified as predictors of good outcome (46, 47). The symptoms of our patient also improved markedly through early treatment. Literature review found that patients respond well to treatment in the third week after onset. Therapy usually combined two or more treatment options, including tumor resection, steroids, IVIg, plasma exchange, cyclophosphamide, infusion of rituximab, or other second-line options. The study case also showed that combination treatment could improve symptoms more than single treatment could. Therapeutic plasma exchange might be an effective rescue therapy to rapidly improve the symptoms of patients with severe steroid/IVIG refractory antibody-associated autoimmune encephalitis (48). It was speculated that early combination therapy would have a better outcome, high survival rate, and low recurrence rate (47). However, long-term follow-up is necessary to observe the improvement of neurological dysfunctions.

In general, patients with anti-NMDA-receptor concurrent with anti-AMPA-receptor encephalitis present with abundant symptoms and often have a history of tumors. Most of them had positive brain MRI results. With timely combination therapy, patients should have a good outcome. The results of the present study indicate the necessity to exclude autoimmune encephalitis for first-episode psychosis and to screen the tumors while anti-NMDA receptor is concurrent with anti-AMPA receptor. This case emphasized the importance of identifying autoimmune encephalitis in patients with schizophrenia and provided insights into the treatment of anti-NMDA-receptor concurrent with anti-AMPA-receptor encephalitis.

## Data Availability Statement

The original contributions presented in the study are included in the article/supplementary material, further inquiries can be directed to the corresponding authors.

## Ethics Statement

The studies involving human participants were reviewed and approved by Ethics Committee of Sichuan Provincial People's Hospital. The patients/participants provided their written informed consent to participate in this study. Written informed consent was obtained from the individual(s) for the publication of any potentially identifiable images or data included in this article.

## Author Contributions

All authors listed have made a substantial, direct, and intellectual contribution to the work and approved it for publication.

## Conflict of Interest

The authors declare that the research was conducted in the absence of any commercial or financial relationships that could be construed as a potential conflict of interest.

## Publisher's Note

All claims expressed in this article are solely those of the authors and do not necessarily represent those of their affiliated organizations, or those of the publisher, the editors and the reviewers. Any product that may be evaluated in this article, or claim that may be made by its manufacturer, is not guaranteed or endorsed by the publisher.
